# Association between the dietary index for gut microbiota and diabetes: the mediating role of phenotypic age and body mass index

**DOI:** 10.3389/fnut.2025.1519346

**Published:** 2025-01-22

**Authors:** Yingxuan Huang, Xiaobo Liu, Chanchan Lin, Xinqi Chen, Yingyi Li, Yisen Huang, Yubin Wang, Xiaoqiang Liu

**Affiliations:** ^1^Department of Gastroenterology, First Hospital of Quanzhou Affiliated to Fujian Medical University, Quanzhou, Fujian, China; ^2^McConnell Brain Imaging Centre, Montreal Neurological Institute, McGill University, Montreal, QC, Canada

**Keywords:** dietary index for gut microbiota, diabetes, phenotypic age, body mass index, mediation analysis, NHANES

## Abstract

**Objectives:**

The global prevalence of diabetes is continuously rising, and the gut microbiota is closely associated with it. The Dietary Index for Gut Microbiota (DI-GM) assesses the impact of diet on the microbiota, but its association with diabetes risk remains unclear. This study aims to investigate the association between DI-GM and the risk of diabetes and analyze the mediating roles of phenotypic age and body mass index (BMI).

**Methods:**

Utilizing data from the National Health and nutrition examination survey (NHANES) 1999–2018, we included 17,444 adults aged 20 years and older. DI-GM (score range: 0–13) was calculated based on dietary recall. Diabetes was diagnosed based on laboratory results and self-reported information. Multivariable logistic regression was used to analyze the association between DI-GM and diabetes, adjusting for relevant covariates. Mediation analysis evaluated the roles of phenotypic age and BMI.

**Results:**

After adjusting for confounders, higher DI-GM scores were significantly associated with a lower risk of diabetes (OR = 0.93, 95% CI = 0.90–0.96, *p* < 0.001). Compared to the group with DI-GM scores of 0–3, those with scores of 5 (OR = 0.76, 95% CI = 0.67–0.86) and ≥ 6 (OR = 0.77, 95% CI = 0.68–0.88) had significantly reduced diabetes risk. Phenotypic age and BMI accounted for 41.02 and 25.57% of the association between DI-GM and diabetes, respectively.

**Conclusion:**

Higher DI-GM scores are associated with a lower risk of diabetes, partially mediated through reduced phenotypic age and BMI.

## 1 Introduction

Diabetes is a metabolic disease characterized by hyperglycemia, primarily classified into type 1 and type 2 diabetes, with type 2 diabetes mellitus (T2DM) accounting for the majority (1). According to the Global Burden of Disease study, the incidence and prevalence of diabetes have significantly increased over the past decades, becoming a major global public health challenge (2). Diabetes not only reduces patients’ quality of life but also increases the risk of complications such as cardiovascular disease, nephropathy, and retinopathy, imposing a heavy economic burden on individuals and society (3) Therefore, exploring effective prevention and management strategies to address the diabetes epidemic is urgently needed.

In recent years, the role of the gut microbiota in metabolic diseases has received widespread attention. Studies have shown that dysbiosis of the gut microbiota is closely associated with insulin resistance, chronic inflammation, and glucose metabolism disorders (4, 5). Therefore, maintaining a healthy gut microbiota may be a potential avenue for preventing and managing diabetes. Diet is a key factor influencing the composition and function of the gut microbiota. Different dietary patterns can significantly alter the diversity and metabolic products of the gut microbiota, thereby affecting the host’s metabolic health (6). Accordingly, Kase et al., based on a review of 106 articles on adult diet and gut microbiota relationships, proposed a new dietary index—the Dietary Index for Gut Microbiota (DI-GM)—to assess the impact of diet on the gut microbiota (7). DI-GM includes 14 dietary components that are beneficial or detrimental to the gut microbiota and effectively reflects the association between dietary quality and gut microbiota diversity.

Moreover, biological age and obesity are important factors influencing diabetes risk. Phenotypic age is an aging indicator based on biomarkers, reflecting an individual’s health status and disease risk (8, 9). Obesity, usually measured by Body Mass Index (BMI), is one of the main risk factors for diabetes (10). Previous studies have shown that dysbiosis of the gut microbiota can accelerate biological aging processes and promote inflammation in adipose tissue, increasing the risk of metabolic diseases (11, 12). Therefore, exploring the association between DI-GM and diabetes, as well as the mediating roles of phenotypic age and BMI, is of significant research value.

However, current research on the relationship between DI-GM and diabetes risk remains limited. To fill this research gap, this study utilized a large representative sample from the National Health and Nutrition Examination Survey (NHANES) to investigate the association between DI-GM and the risk of diabetes. Additionally, we analyzed the mediating roles of phenotypic age and BMI in this association. Our study contributes to a deeper understanding of the complex relationships among diet, gut microbiota, biological age, and diabetes, providing new scientific evidence for the prevention and intervention of diabetes.

## 2 Methods

### 2.1 Data source

This study used data from the NHANES 1999–2018. NHANES is a continuous cross-sectional survey based on the non-institutionalized population in the United States, collecting participants’ health, nutrition, and demographic data through a multistage probability sampling method. The data used were derived from public files and were approved by the National Center for Health Statistics Ethics Review Board, with all participants providing written informed consent. This study follows the Strengthening the Reporting of Observational Studies in Epidemiology (STROBE) reporting guidelines.

### 2.2 Study design and population

**Figure 1:**
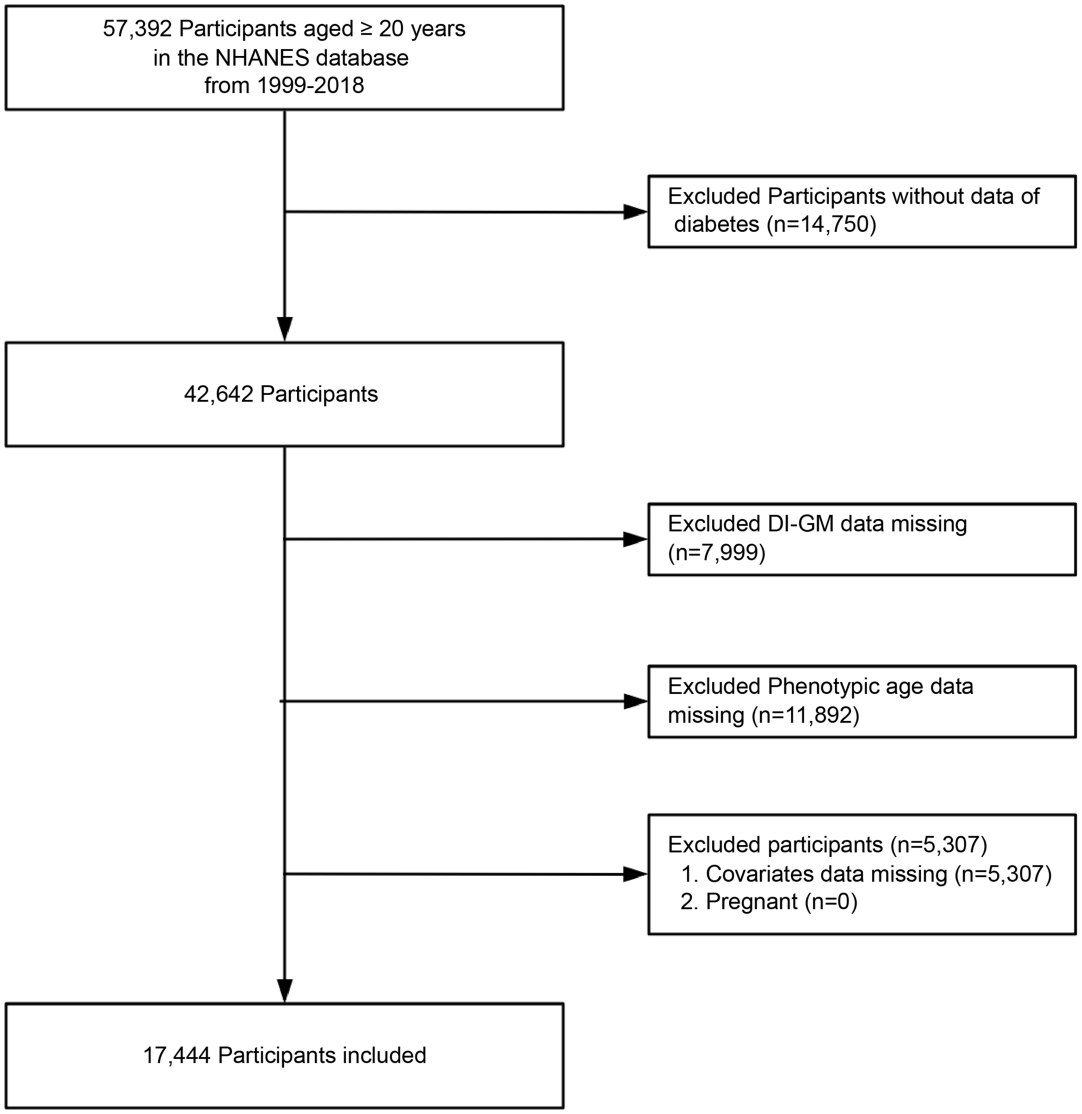
Flow chart of the screening of the NHANES 1999–2018 participants.

Participants were adults aged 20 years and older who took part in NHANES from 1999 to 2018. During initial screening, individuals lacking diabetes diagnosis data, components of the DI-GM, phenotypic age, BMI data, and covariates were excluded. A total of 17,444 eligible participants were included in the analysis, of whom 3,334 were diagnosed with diabetes (Figure 1).

### 2.3 Definition of diabetes

Diabetes was diagnosed based on laboratory test results and self-reported information provided in NHANES. Diagnostic criteria included any of the following: physician diagnosis of diabetes, glycated hemoglobin (HbA1c) level ≥ 6.5%, fasting blood glucose level ≥ 7.0 mmol/L, random or 2-h oral glucose tolerance test (OGTT) blood glucose level ≥ 11.1 mmol/L, or use of diabetes medication/insulin. Participants meeting any of these criteria were classified as having diabetes (13).

### 2.4 Assessment of the dietary index for gut microbiota

The DI-GM is a novel dietary quality assessment index based on the relationship between diet and gut microbiota, aiming to reflect the potential impact of dietary patterns on gut microbiota diversity and identify dietary characteristics that help maintain a healthy gut microbiota (7). The dietary data from NHANES were obtained through a 24-h recall method (Automated Multiple-Pass Method, AMPM) developed by the United States Department of Agriculture (USDA) (14). This standardized interview procedure, administered by professionally trained interviewers, captures all foods and beverages consumed within the previous 24 h. During data collection, NHANES implemented uniform training for interviewers and employed standardized protocols and tools, thereby minimizing interviewer bias and recall bias from participants (15). For the calculation of DI-GM and the analysis of other diet-related variables, we took the average of two independent 24-h dietary recall interviews for each participant. DI-GM consists of 14 food or nutrient components, including 10 considered beneficial for gut microbiota diversity—avocado, broccoli, chickpeas, coffee, cranberries, fermented dairy products, fiber, green tea (this component may be omitted in some analyses due to NHANES not specifically recording green tea consumption), soy, and whole grains—and 4 components considered detrimental to gut microbiota diversity—red meat, processed meats, refined grains, and high-fat diets (≥40% of total energy from fat). Scoring is based on sex-specific median intake levels: for beneficial components, a score of 1 is assigned if intake is above the median and 0 if below; for detrimental components, a score of 1 is assigned if intake is below the median and 0 if above (for high-fat diets, a score of 1 is assigned if fat intake is less than 40% of total energy). The components along with scoring criteria for the DI-GM can be found in Supplementary Table 1. The total DI-GM score ranges from 0 to 13, with beneficial components contributing 0 to 9 points and detrimental components contributing 0 to 4 points. Higher scores indicate greater potential dietary benefits to the gut microbiota. Based on previous research, DI-GM scores were divided into four categories based on quartiles: 0–3, 4, 5, and ≥ 6 points (16).

### 2.5 Definition of phenotypic age and BMI

Phenotypic age was calculated based on an algorithm involving 10 clinical biomarkers, including chronological age (CA), albumin, creatinine, blood glucose, C-reactive protein, lymphocyte percentage, mean corpuscular volume, red cell distribution width, alkaline phosphatase, and white blood cell count (17). BMI was calculated by dividing weight in kilograms by height in meters squared.

### 2.6 Covariates

Based on previous research and clinical judgment, multiple potential confounding variables were considered, including age, gender, race, marital status, education level, poverty income ratio (PIR), physical activity, smoking status, alcohol intake, cardiovascular disease (CVD), hypertension, and hyperlipidemia (18, 19). Specific definitions are as follows: Age was treated as a continuous variable, recording participants’ actual age. Gender recorded participants’ gender. Race was categorized as non-Hispanic White, others (non-Hispanic Black, Mexican American, other Hispanic, and other races). Marital status was categorized as married/living with a partner and unmarried/other (including widowed, divorced, or separated). PIR was divided into three categories: 1–1.3, 1.31–3.50, and > 3.50 (20). Education level was classified as less than high school, high school or equivalent, and more than high school. Smoking status was categorized as never smokers (smoked less than 100 cigarettes in their lifetime), former smokers (smoked more than 100 cigarettes but are currently non-smokers), and current smokers (smoked more than 100 cigarettes and currently smoke occasionally or daily) (21). Participants were categorized according to alcohol intake as never (< 12 drinks in their lifetime), former (≥ 12 drinks in 1 year and did not drink last year or did not drink last year but drank ≥12 drinks in their lifetime), or current drinkers (including heavy alcohol use [≥3 drinks per day for females, ≥4 drinks per day for males, or binge drinking ≥4 drinks on the same occasion for females or ≥ 5 drinks for males on 5 or more days per month], moderate alcohol use [≥2 drinks per day for females, ≥3 drinks per day for males, or binge drinking ≥2 days per month], and mild alcohol use [≤1 drink per day for females, ≤2 drinks per day for males]) (22). Physical activity time was a continuous variable indicating the time spent in walking, cycling, work, and recreational activities per week, categorized into three levels: inactive (0 MET-min/week), insufficiently active (1–599 MET-min/week), and sufficiently active (≥600 MET-min/week) (23). CVD: Self-reported diagnosis of coronary heart disease, angina, stroke, myocardial infarction, or congestive heart failure. Hypertension was defined by an average systolic blood pressure ≥ 140 mmHg and/or diastolic blood pressure ≥ 90 mmHg, self-reported diagnosis, or use of antihypertensive medications (21); hyperlipidemia was defined as meeting any one of the following criteria: (1) use of lipid-lowering medications; (2) hypertriglyceridemia (≥150 mg/dL); (3) hypercholesterolemia (total cholesterol ≥200 mg/dL, or LDL ≥130 mg/dL, or HDL <40 mg/dL).

### 2.7 Statistical analysis

All statistical analyses were performed using R statistical software and Free Statistics software. Statistical significance was defined as a two-sided *p*-value < 0.05. Continuous variables were described using means and standard deviations (SD), and categorical variables were expressed as percentages. Group differences were compared using chi-square tests, two-sample independent t-tests, and Mann–Whitney U tests.

To evaluate the association between DI-GM and the risk of diabetes, multivariable logistic regression models were constructed to calculate odds ratios (OR) and their 95% confidence intervals (CI). The models included: Model 1: Crude model without adjustment for any covariates. Model 2: Fully adjusted model, adjusting for all potential confounders listed above. Additionally, DI-GM was divided into four categories (0–3, 4, 5, ≥6) to explore the effect of DI-GM grouping on diabetes risk. Furthermore, we performed an analysis for each individual components of DI-GM to assess their independent associations with diabetes risk. Restricted cubic spline (RCS) analysis was employed to assess the potential nonlinear relationship between DI-GM and diabetes, setting four knots at the 5th, 35th, 65th, and 95th percentiles of DI-GM scores. Subgroup analyses were conducted based on variables such as age, gender, physical activity, smoking status, alcohol intake, CVD, hypertension, and hyperlipidemia to assess the consistency of the association across different populations.

Sensitivity analyses included: (1) Multiple Imputation: Missing data were handled using the multiple imputation method with chained equations (MICE), generating five imputed datasets. Logistic regression analyses were repeated on these datasets. (2) Propensity Score Matching (PSM): A 1: 1 PSM was conducted using “diabetes status” (presence or absence of diabetes) as the primary matching variable to address potential confounding. Logistic regression analyses were then conducted on the matched sample. (3) Following the NHANES analytical guidelines, we accounted for the complex sampling design and incorporated mobile examination center (MEC) sample weights into our analysis to address batch effects, including variations in data collection time periods and geographic distribution (24). This adjustment ensures that our findings are representative of the U.S. population and accounts for potential biases arising from differences in sampling methods across survey cycles. To evaluate the association between DI-GM and the risk of diabetes, we constructed multivariable logistic regression models to calculate ORs with their corresponding 95% CIs. Detailed information regarding the weighted analysis can be found in the Supplementary materials. Mediation analyses explored the roles of phenotypic age and BMI using the Sobel test and bootstrap method with 1,000 simulations to calculate 95% CIs of the mediation effect. The mediation effect was expressed as the proportion mediated.

## 3 Results

### 3.1 Participant characteristics

As shown in Table 1, the study included 17,444 participants from NHANES 1999–2018, of whom 3,334 were diagnosed with diabetes and 14,110 did not have diabetes. The average age was 50.62 years (SD = 17.59). Compared to non-diabetic individuals, participants with diabetes were older (61.26 vs. 48.11 years, *p* < 0.001) and had a higher proportion of males (51.83% vs. 48.86%, *p* = 0.002). Significant differences were also observed in race, income ratio, education level, smoking and alcohol status, physical activity level, and comorbidities such as CVD, hypertension, and hyperlipidemia (*p* < 0.05).

**Table 1:** Characteristics of the NHANES 1999–2018 participants.

Variables	Total (*n* = 17,444)	Without diabetes	Diabetes	*p-value*
Number of participants	17,444	14,110	3,334	
Age, Mean ± SD	50.62 ± 17.59	48.11 ± 17.52	61.26 ± 13.41	< 0.001
Gender, n (%)				0.002
Male	8,622 (49.43)	6,894 (48.86)	1728 (51.83)	
Female	8,822 (50.57)	7,216 (51.14)	1,606 (48.17)	
Race, n (%)				< 0.001
Non-Hispanic White	8,298 (47.57)	6,945 (49.22)	1,353 (40.58)	
Others	9,146 (52.43)	7,165 (50.78)	1981 (59.42)	
Marital status, n (%)				0.313
Married/ Living with partner	10,765 (61.71)	8,733 (61.89)	2032 (60.95)	
Never married/Other	6,679 (38.29)	5,377 (38.11)	1,302 (39.05)	
PIR group, n (%)				< 0.001
1–1.3	4,963 (28.45)	3,904 (27.67)	1,059 (31.76)	
1.31–3.50	6,911 (39.62)	5,493 (38.93)	1,418 (42.53)	
>3.50	5,570 (31.93)	4,713 (33.40)	857 (25.70)	
Education level, n (%)				< 0.001
Less than high school	4,032 (23.11)	2,988 (21.18)	1,044 (31.31)	
High school or equivalent	4,144 (23.76)	3,312 (23.74)	832 (24.96)	
Above high school	9,268 (53.13)	7,810 (55.35)	1,458 (43.73)	
Smoking status, n (%)				< 0.001
Never	9,374 (53.74)	7,716 (54.68)	1,658 (49.73)	
Former	4,567 (26.18)	3,416 (24.21)	1,151 (34.52)	
Current	3,503 (20.08)	2,978 (21.11)	525 (15.75)	
Alcohol intake, n (%)				< 0.001
Never	2,196 (12.59)	1,679 (11.90)	517 (15.51)	
Former	3,431 (19.67)	2,417 (17.13)	1,014 (30.41)	
Current	11,817 (67.74)	10,014 (70.97)	1803 (54.08)	
Physical activity, n (%)				< 0.001
Inactive	4,432 (25.41)	3,156 (22.37)	1,276 (38.27)	
Insufficiently active	3,335 (19.12)	2,713 (19.23)	622 (18.66)	
Sufficiently active	9,677 (55.47)	8,241 (58.41)	1,436 (43.07)	
CVD, n (%)				< 0.001
No	15,404 (88.31)	12,926 (91.61)	2,478 (74.33)	
Yes	2040 (11.69)	1,184 (8.39)	856 (25.67)	
Hypertension, n (%)				< 0.001
No	9,807 (56.22)	8,891 (63.01)	916 (27.47)	
Yes	7,637 (43.78)	5,219 (36.99)	2,418 (72.53)	
Hyperlipidemia, n (%)				< 0.001
No	4,716 (27.04)	4,295 (30.44)	421 (12.63)	
Yes	12,728 (72.96)	9,815 (69.56)	2,913 (87.37)	
DI-GM score, Mean ± SD	4.52 ± 1.52	4.54 ± 1.52	4.46 ± 1.52	0.01
DI-GM group, n (%)				0.234
0–3	4,446 (25.49)	3,553 (25.18)	893 (26.78)	
4	4,363 (25.01)	3,529 (25.01)	834 (25.01)	
5	4,201 (24.08)	3,426 (24.28)	775 (23.25)	
≥6	4,434 (25.42)	3,602 (25.53)	832 (24.96)	
Beneficial to gut microbiota	2.00 (1.00, 3.00)	2.00 (1.00, 3.00)	2.00 (1.00, 3.00)	0.778
Unfavorable to gut microbiota	2.31 ± 1.03	2.32 ± 1.02	2.27 ± 1.04	0.005
BMI (kg/m^2^), Mean ± SD	29.42 ± 6.88	28.69 ± 6.52	32.54 ± 7.46	< 0.001
Phenotypic age, Mean ± SD	50.13 ± 21.0	45.86 ± 19.40	68.18 ± 17.64	< 0.001

DI-GM, dietary index for gut microbiota; BMI, Body Mass Index; PIR, poverty income ratio; CVD, cardiovascular disease.

### 3.2 Association between DI-GM and diabetes

As shown in Table 2, multivariable logistic regression indicated that higher DI-GM scores were significantly associated with a lower risk of diabetes. In the crude model, each unit increase in DI-GM was associated with a 3% decrease in the odds of having diabetes (OR = 0.97, 95% CI = 0.94–0.99, *p* = 0.01); in the adjusted model, the association was more pronounced (OR = 0.93, 95% CI = 0.90–0.96, *p* < 0.001). Compared to the group with DI-GM scores of 0–3, those with scores of 5 (OR = 0.76, 95% CI = 0.67–0.86, *p* < 0.001) and ≥ 6 (OR = 0.77, 95% CI = 0.68–0.88, *p* < 0.001) were significantly associated with lower odds of having diabetes.

**Table 2:** Association between DI-GM and diabetes.

Characteristics	Diabetes			
	Crude model		Adjusted model	
	OR (95% CI)	*p-*value	OR (95% CI)	*p-*value
DI-GM	0.97 (0.94–0.99)	0.01	0.93 (0.90–0.96)	<0.001
DI-GM group				
0–3	Ref		Ref	
4	0.94 (0.85–1.04)	0.252	0.86 (0.77–0.97)	0.014
5	0.90 (0.81–1.00)	0.054	0.76 (0.67–0.86)	<0.001
≥6	0.92 (0.83–1.02)	0.116	0.77 (0.68–0.88)	<0.001
Trend test		0.081		<0.001
Beneficial to gut microbiota	0.99 (0.96–1.02)	0.397	1.00 (0.97–1.04)	0.794
Unfavorable to gut microbiota	0.95 (0.91–0.98)	0.005	0.85 (0.82–0.89)	<0.001

DI-GM, dietary index for gut microbiota; PIR, poverty income ratio; CVD, cardiovascular disease; CI, Confidence interval; OR, Odd Ratio.

**Figure 2:**
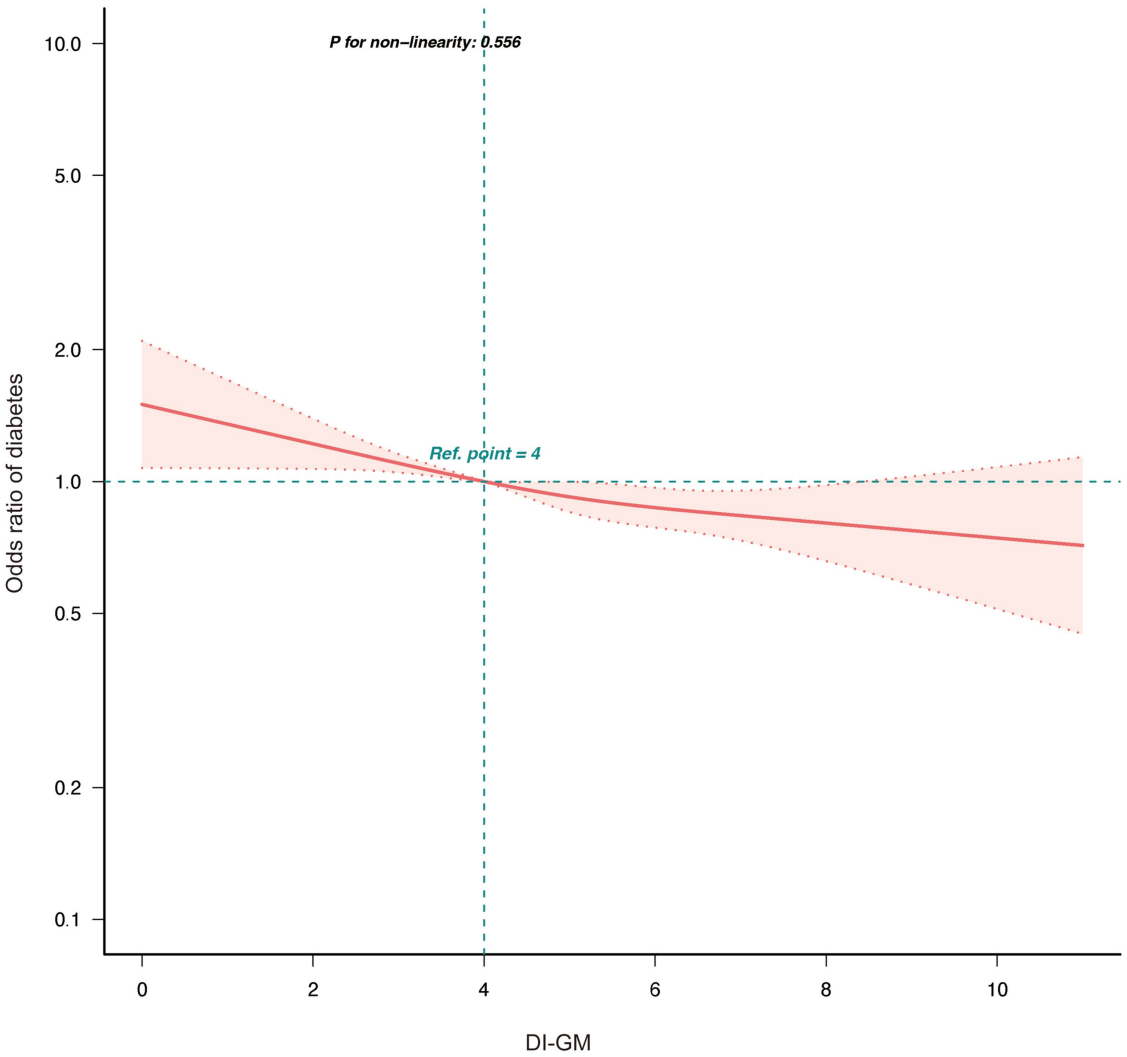
Association between DI-GM and diabetes in NHANES 1999–2018 participants by RCS. DI-GM, dietary index for gut microbiota; PIR, poverty income ratio; CVD, cardiovascular disease; RCS, restricted cubic spline. The model was adjusted for age, gender, race, marital status, education level, PIR, physical activity, smoking status, Alcohol intake, CVD, Hypertension, and Hyperlipidemia.

**Figure 3:**
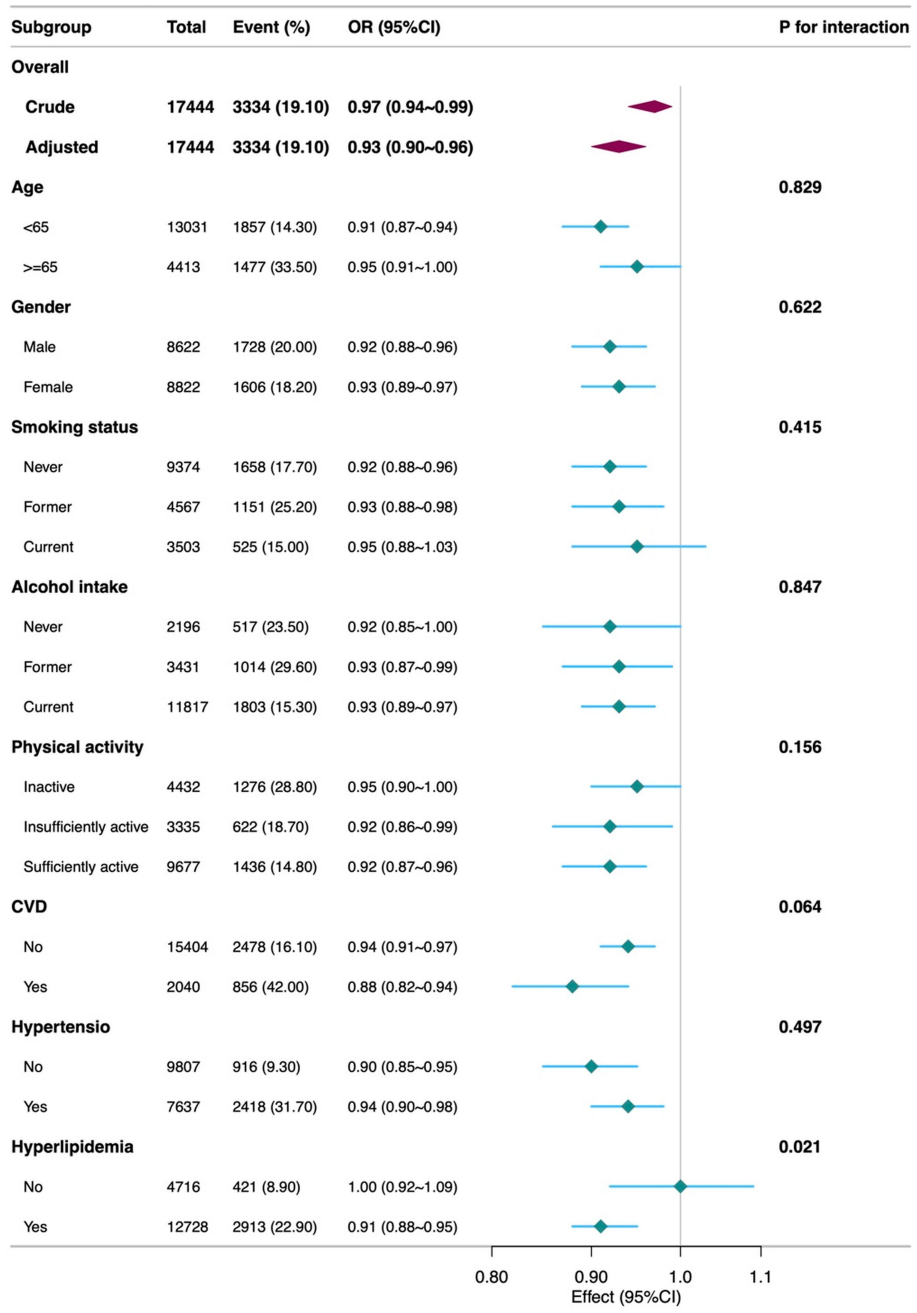
Subgroup analyses of the Association between DI-GM and diabetes among participants. DI-GM, dietary index for gut microbiota; PIR, poverty income ratio; CVD, cardiovascular disease; CI, Confidence interval; OR, Odd Ratio. The model was adjusted for age, gender, race, marital status, education level, PIR, physical activity, smoking status, Alcohol intake, CVD, Hypertension, and Hyperlipidemia.

Further analysis showed that higher scores for detrimental dietary components were significantly associated with increased diabetes risk (OR = 0.85, 95% CI = 0.82–0.89, *p* < 0.001), whereas scores for beneficial components were not significantly associated (*p* = 0.794). Analysis of individual components of DI-GM revealed the following results: Among the beneficial components, whole grains (OR = 1.12, 95% CI = 1.02–1.22, *p* = 0.015) were associated with a higher risk of diabetes, while coffee (OR = 0.89, 95% CI = 0.82–0.98, *p* = 0.012) was linked to a lower risk in the adjusted model. Other beneficial components, including fiber, fermented dairy, avocados, and soybeans, did not demonstrate significant associations after adjustment for confounders (Supplementary Table 2). RCS analysis (Figure 2) indicated a linear association between DI-GM and diabetes risk (*p* = 0.556). Subgroup analyses demonstrated that the negative association between DI-GM and diabetes was significant in most subgroups, indicating the applicability and robustness of DI-GM across different populations (Figure 3).

### 3.3 Sensitivity analysis

Multiple imputation results showed that the negative association between DI-GM and diabetes remained significant in the adjusted model (OR = 0.93, 95% CI = 0.89–0.96, *p* < 0.001). PSM analysis results were consistent with multiple imputation, further supporting the robustness of the main findings (Table 3). In Supplementary Table 3, the weighted analysis confirmed the stability of the association between DI-GM and diabetes risk.

**Table 3:** Sensitivity analyses.

	Diabetes			
	Crude model		Adjusted model	
	OR (95% CI)	*p-*value	OR (95% CI)	*p-*value
Multiple imputations of missing data	0.94 (0.91–0.97)	<0.001	0.93 (0.89–0.96)	<0.001
Propensity score matching	0.98 (0.96–1.00)	0.046	0.93 (0.90–0.96)	<0.001

DI-GM, dietary index for gut microbiota; PIR, poverty income ratio; CVD, cardiovascular disease; CI, Confidence interval; OR, Odd Ratio.

### 3.4 Mediation analysis

**Figure 4:**
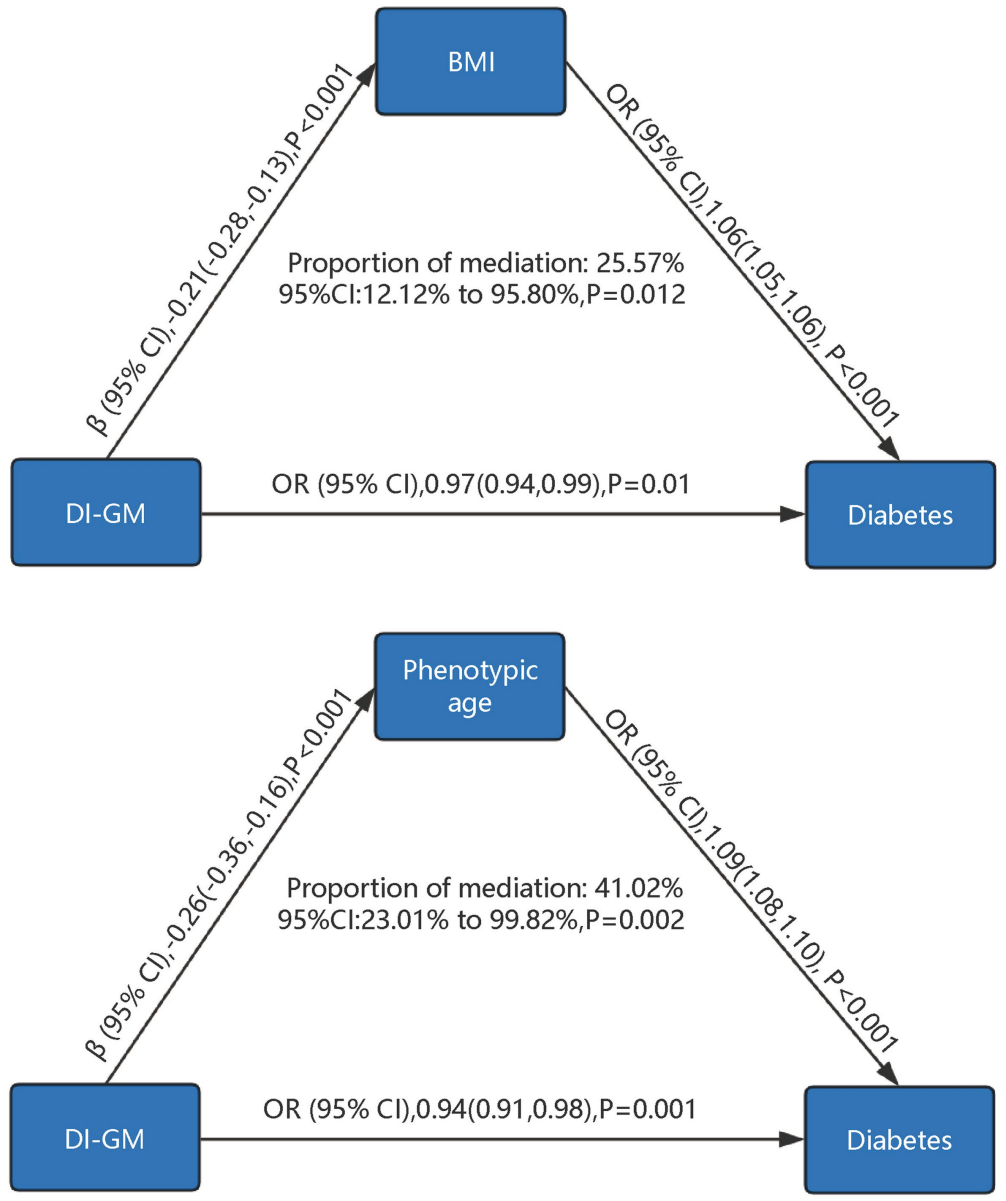
Mediation analysis of phenotypic age and body mass index in the association between DI-GM and diabetes. DI-GM, dietary index for gut microbiota; BMI, Body Mass Index; PIR, poverty income ratio; CVD, cardiovascular disease; CI, Confidence interval; OR, Odd Ratio. The models were adjusted for age, gender, race, marital status, education level, poverty-to-income ratio (PIR), physical activity, smoking status, alcohol intake, cardiovascular disease (CVD), hypertension, hyperlipidemia, as well as BMI and phenotypic age, with mutual adjustment performed between BMI and phenotypic age to account for their potential interdependence in the analysis.

As shown in Figure 4, mediation analysis explored the mediating roles of BMI and phenotypic age. Higher DI-GM scores were associated with lower BMI (*β* = −0.21, 95% CI = −0.28 to −0.13, *p* < 0.001) and lower phenotypic age (*β* = −0.26, 95% CI = −0.36 to −0.16, *p* < 0.001). Increases in BMI (OR = 1.06, 95% CI = 1.05–1.06, *p* < 0.001) and phenotypic age (OR = 1.09, 95% CI = 1.08–1.10, *p* < 0.001) significantly increased diabetes risk. BMI accounted for 25.57% (95% CI = 12.12–95.80%, *p* = 0.012) and phenotypic age for 41.02% (95% CI = 23.01–99.82%, *p* = 0.002) of the association between DI-GM and diabetes.

## 4 Discussion

This study systematically evaluated the association between DI-GM and diabetes risk and explored the mediating roles of phenotypic age and BMI. Our results showed that higher DI-GM scores were significantly associated with a lower risk of diabetes, partially mediated through inverse in phenotypic age and BMI. This finding underscores the complex relationships among dietary patterns, gut microbiota, biological aging, weight control, and diabetes.

Previous studies have shown that dysbiosis of the gut microbiota is closely associated with insulin resistance, chronic inflammation, and glucose metabolism disorders (25). Diet is a key factor influencing the gut microbiota; different dietary patterns can lead to significant changes in gut microbiota diversity and metabolic products (6). Our findings support these views, indicating that optimizing diet to promote a healthy gut microbiota can reduce the risk of diabetes. Notably, we found that higher scores for dietary components detrimental to the gut microbiota (such as red meat, processed meats, refined grains, and high-fat diets) were significantly associated with increased diabetes risk. This is consistent with previous studies; excessive consumption of these foods has been shown to reduce gut microbiota diversity, promote the proliferation of harmful bacteria, and induce inflammatory responses and metabolic disorders (26, 27). For example, high-fat diets can decrease the proportion of Bacteroidetes and increase the proportion of Firmicutes, affecting energy metabolism (28). In contrast, foods rich in dietary fiber and phytochemicals (such as whole grains, legumes, and fruits) help increase beneficial gut bacteria like *Bifidobacterium* and *Lactobacillus*, produce short-chain fatty acids, and improve insulin sensitivity (29).

However, scores for beneficial dietary components, except for coffee, were not significantly associated with a lower risk of diabetes. This may be due to various factors. First, the impact of diet on the gut microbiota is complex and individualized; dietary habits, genetic background, and lifestyle factors may influence results (30). Second, beneficial effects may require a longer duration to manifest, which our cross-sectional design could not capture (31). Additionally, dietary intake data based on 24-h recalls may not fully reflect long-term dietary patterns.

The inclusion of phenotypic age and BMI in the mediation analysis in this study was primarily based on an exploratory perspective, aiming to identify potential mediating pathways between the DI-GM and diabetes. This approach also seeks to provide theoretical support and directions for future longitudinal studies or interventional trials. Specifically, phenotypic age, as a metric that quantifies overall health status and the degree of biological aging, was selected because accelerated biological aging is commonly observed in patients with diabetes (32). Moreover, biological aging is closely associated with alterations in gut microbiota composition and changes in dietary behaviors (33). BMI, a widely used indicator of obesity, was included due to its established role as a critical risk factor for diabetes (10). Poor dietary habits and gut microbiota dysbiosis may contribute to obesity and insulin resistance, thereby increasing the risk of developing diabetes (34). Thus, phenotypic age and BMI are important mediators that may play a key role in the “diet-gut microbiota” pathway and its association with diabetes. The mediation analysis showed that phenotypic age and BMI had significant mediating effects in the association between DI-GM and diabetes. This implies that higher DI-GM scores may reduce diabetes risk by lowering biological age and controlling weight. Phenotypic age reflects biological aging, and accelerated aging is associated with increased risks of diabetes and other chronic diseases (35). The gut microbiota can influence aging processes by regulating inflammatory responses, oxidative stress, and metabolic functions (36, 37). For instance, dietary fiber enhances the growth of butyrate-producing bacteria such as *Faecalibacterium* and *Roseburia*, which produce SCFAs that reduce inflammation and oxidative stress, thereby delaying aging (38). Furthermore, the gut microbiota is closely related to obesity, affecting energy intake and fat storage, thereby influencing BMI (39, 40). Improving diet to promote a healthy gut microbiota may help delay aging, control weight, and reduce diabetes risk.

Strengths of this study include the use of the large, nationally representative NHANES dataset, enhancing external validity. We adjusted for multiple potential confounders to reduce bias and validated the robustness of results through sensitivity analyses and PSM. However, limitations exist. First, the cross-sectional design cannot establish causality. Longitudinal studies and randomized controlled trials are needed to confirm causal associations. Second, dietary intake data were self-reported, possibly introducing recall bias. Future studies may use more objective dietary assessment methods. Third, unmeasured confounders, such as genetic factors and direct gut microbiota sequencing data, may influence results. Additionally, due to NHANES limitations, we could not account for green tea consumption, potentially underestimating DI-GM scores.

Future research should explore mechanistic relationships among diet, gut microbiota, biological age, and metabolic diseases. Understanding how specific dietary components affect the gut microbiota can aid in developing personalized dietary interventions. Considering individual variability, studies should focus on specific effects in different populations.

## 5 Conclusion

In summary, this study found that a higher DI-GM score was associated with a lower prevalence of diabetes, partly mediated by reductions in phenotypic age and BMI. Although we did not directly measure changes in the gut microbiome, these findings highlight the importance of dietary patterns in metabolic health. Future research and interventions leveraging the DI-GM may help inform strategies to reduce the burden of diabetes.

## Data Availability

Publicly available datasets were analyzed in this study. All data entered into the analysis were from NHANES, which is publicly accessible to all.
