# Supplementary material for: Association between the dietary index for gut microbiota and diabetes: the mediating role of phenotypic age and body mass index
Source: Front Nutr. 2025 Jan 22;12:1519346. doi: 10.3389/fnut.2025.1519346 (PMC11794117; doi:10.3389/fnut.2025.1519346)
Supplement: Supplementary file 1 [file Table_1.DOCX]

**Supplementary Table 1** **Components and scoring criteria of DI-GM in NHANES.**

| **Components of**  **DI-GM** | **Food items included in NHANES** | **Scoring criteria** |
| --- | --- | --- |
| **Beneficial to gut microbiota** | Avocados, Broccoli, Chickpeas, Coffee, Cranberries, Fermented dairy (including yogurt, cheese, kefir, sour cream, buttermilk), Fiber, Soybean (including Soy milk, Tofu), Whole grains | Score 1 - Consumption ≥ sex-specific median  Score 0 - Otherwise |
| **Unfavorable to gut microbiota** | Refined grains, Processed meat, Red meat | Score 0 - Consumption ≥ sex-specific median  Score 1 - Otherwise |
|  | High-fat diet (% energy) | Score 0 - Consumption ≥ 40%  Score 1 - Otherwise |

Abbreviations: DI-GM, dietary index for gut microbiota; NHANES, National Health and Nutrition Examination Survey.

**Supplementary Table 2 Association between scores of individual components of the DI-GM and diabetes**

| **Characteristics** | **Diabetes** | | | | |
| --- | --- | --- | --- | --- | --- |
|  | **Crude model** | |  | **Adjusted model** | |
|  | **OR (95% CI)** | ***P* -value** |  | **OR (95% CI)** | ***P* -value** |
| **Beneficial to gut microbiota** |  |  |  |  |  |
| Avocados | 0.78 (0.61~1.00) | 0.050 |  | 0.94 (0.72~1.22) | 0.637 |
| Broccoli | 0.90 (0.79~1.03) | 0.132 |  | 1.00 (0.87~1.16) | 0.975 |
| Chickpeas | 1.29 (0.75~2.22) | 0.365 |  | 1.60 (0.89~2.90) | 0.118 |
| Coffee | 1.25 (1.16~1.35) | <0.001 |  | 0.89 (0.82~0.98) | 0.012 |
| Cranberries | 0.69 (0.47~1.01) | 0.054 |  | 0.70 (0.47~1.05) | 0.087 |
| Fermented dairy | 0.74 (0.69~0.80) | <0.001 |  | 1.07 (0.98~1.16) | 0.130 |
| Fiber | 0.93 (0.86~1.00) | 0.044 |  | 1.04 (0.95~1.13) | 0.443 |
| Soybean | 0.68 (0.49~0.95) | 0.024 |  | 0.79 (0.55~1.12) | 0.183 |
| Whole grains | 1.18 (1.10~1.28) | <0.001 |  | 1.12 (1.02~1.22) | 0.015 |
| **Unfavorable to gut microbiota** |  |  |  |  |  |
| Refined grains | 1.26 (1.16~1.35) | <0.001 |  | 1.00 (0.91~1.09) | 0.921 |
| Processed meat | 0.83 (0.77~0.89) | <0.001 |  | 0.81 (0.74~0.88) | <0.001 |
| Red meat | 1.01 (0.94~1.09) | 0.820 |  | 0.94 (0.86~1.02) | 0.132 |
| High-fat diet | 0.72 (0.66~0.78) | <0.001 |  | 0.67 (0.61~0.73) | <0.001 |

Abbreviations: DI-GM, dietary index for gut microbiota; PIR, poverty income ratio; CVD, cardiovascular disease; CI, Confidence interval; OR, Odd Ratio.

^a^.The crude model was not adjusted for any covariates, while the adjusted model was adjusted for age, gender, race, marital status, education level, PIR, physical activity, smoking status, Alcohol intake, CVD, Hypertension, and Hyperlipidemia.

**Supplementary Table 3 Weighted analysis of the association between DI-GM and diabetes** **(further adjusted for survey cycle)**

| **Characteristics** | **Diabetes** | | | | |
| --- | --- | --- | --- | --- | --- |
|  | **Crude model** | |  | **Adjusted model** | |
|  | **OR (95% CI)** | ***P* -value** |  | **OR (95% CI)** | ***P* -value** |
| DI-GM | 0.94 (0.91~0.97) | <0.001 |  | 0.92 (0.89~0.95) | <0.001 |
| DI-GM group |  |  |  |  |  |
| 0-3 | Ref |  |  | Ref |  |
| 4 | 0.93 (0.81~1.07) | 0.278 |  | 0.87 (0.74~1.04) | 0.126 |
| 5 | 0.92 (0.81~1.04) | 0.170 |  | 0.80 (0.70~0.92) | 0.002 |
| ≥6 | 0.81 (0.69~0.93) | 0.005 |  | 0.73 (0.61~0.86) | <0.001 |
| Trend test |  | 0.006 |  |  | <0.001 |
| Beneficial to gut microbiota | 0.97 (0.93~1.02) | 0.206 |  | 0.98 (0.94~1.03) | 0.429 |
| Unfavorable to gut microbiota | 0.90 (0.86~0.95) | <0.001 |  | 0.86 (0.81~0.91) | <0.001 |

Abbreviations: DI-GM, dietary index for gut microbiota; PIR, poverty income ratio; CVD, cardiovascular disease; CI, Confidence interval; OR, Odd Ratio.

^a^.The crude model was not adjusted for any covariates, while the adjusted model was adjusted for survey cycle，age, gender, race, marital status, education level, PIR, physical activity, smoking status, Alcohol intake, CVD, Hypertension, and Hyperlipidemia.

^b^.The DI-GM ranges from 0–13 (including beneficial to gut microbiota [ranges from 0–9] and unfavorable to gut microbiota [ranges from 0–4]) and grouped according to 0–3, 4, 5, and ≥ 6.

Note: Analyses were conducted on a total sample size of 95,993,479 participants after weighted analysis. The adjustment models included the NHANES “survey cycle” as a covariate and applied sample weight correction, primarily to control for potential effects of regional and temporal batch variations on the results.

Weighted analysis is a statistical method that assigns different weights to observations in a sample to reflect their importance and representativeness within the overall dataset. This technique enhances the accuracy and efficiency of estimates and allows for a deeper understanding of population characteristics and relationships between variables. By adjusting sample weights, weighted analysis corrects estimation biases, leading to more accurate results. Additionally, it optimizes estimation efficiency by assigning different weights to observations based on their relative importance, ensuring that the sample is representative and leading to more accurate population estimates (https://wwwn.cdc.gov/nchs/nhanes/analyticguidelines.aspx#estimation-and-weighting-procedures). Detailed information and methodology regarding NHANES weighting can be found in the NHANES guidelines on their website. Following these guidelines, we incorporated the complex sampling design and mobile examination center sample weights into our study, ensuring that our data sample represents a large and diverse U.S. adult population. The sampling weights were determined as follows: for the 1999–2002 period, weights were calculated as 2/8 × 4-year MEC weight, while for the 2003–2010 and 2015–2018 periods, weights were 1/8 × 2year MEC weight. It is important to note that data for CRP, which are essential for calculating phenotypic age, were unavailable for the 2011–2014 period.

**Supplementary Table 4 Association between DI-GM and diabetes (further adjusted for total energy intake,** **carbohydrate**

**, sugars, dietary fiber, protein)**

| **Characteristics** | **Diabetes** | | | | |
| --- | --- | --- | --- | --- | --- |
|  | **Crude model** | |  | **Adjusted model** | |
|  | **OR (95% CI)** | ***P* -value** |  | **OR (95% CI)** | ***P* -value** |
| DI-GM | 0.97 (0.94~0.99) | 0.01 |  | 0.94 (0.90~0.97) | <0.001 |
| DI-GM group |  |  |  |  |  |
| 0-3 | Ref |  |  | Ref |  |
| 4 | 0.94 (0.85~1.04) | 0.252 |  | 0.88 (0.78~0.99) | 0.014 |
| 5 | 0.90 (0.81~1.00) | 0.054 |  | 0.78 (0.69~0.89) | <0.001 |
| ≥6 | 0.92 (0.83~1.02) | 0.116 |  | 0.80 (0.70~0.92) | 0.001 |
| Trend test |  | 0.081 |  |  | <0.001 |
| Beneficial to gut microbiota | 0.99 (0.96~1.02) | 0.397 |  | 1.01 (0.97~1.06) | 0.550 |
| Unfavorable to gut microbiota | 0.95 (0.91~0.98) | 0.005 |  | 0.85 (0.81~0.89) | <0.001 |

Abbreviations: DI-GM, dietary index for gut microbiota; PIR, poverty income ratio; CVD, cardiovascular disease; CI, Confidence interval; OR, Odd Ratio.

^a^.The crude model was not adjusted for any covariates, while the adjusted model was adjusted for age, gender, race, marital status, education level, PIR, physical activity, total energy intake, carbohydrate, sugars, dietary fiber, protein, smoking status, Alcohol intake, CVD, Hypertension, and Hyperlipidemia.

^b^.The DI-GM ranges from 0–13 (including beneficial to gut microbiota [ranges from 0–9] and unfavorable to gut microbiota [ranges from 0–4]) and grouped according to 0–3, 4, 5, and ≥ 6.

Note: Analyses were conducted on a total sample size of 17,444 participants. While weight correction was not applied, the model was further adjusted for dietary-related variables, including total energy intake, carbohydrate, sugars, dietary fiber, and protein. This table provides a more in-depth assessment of the association between DI-GM and diabetes, accounting for potential confounding effects of these dietary variables.

**The calculation formula for phenotypic age**

$$Phenotypic age = 141.50 + \frac{Ln[-0.00553 \times Ln(exp(\frac{-1.51714 \times\mathrm{ex}p \left( xb \right)}{0.0076927}))]}{0.09165}$$

where *xb* = − 19.907 − 0.0336 × Albumin (g/L) + 0.0095 × Creatinine (μmol/L) + 0.1953 × Glucose (mmol/L) + 0.0954 × LnCRP (mg/dL) − 0.0120 × Lymphocyte Percent (%) + 0.0268 × Mean Cell Volume (fL) + 0.3306 × Red Cell Distribution Width (%) + 0.00188 × Alkaline Phosphatase (U/L) + 0.0554 × White Blood Cell Count (1000 cells/ μL) + 0.0804 × Chronological Age (years)^14^.
